# Coexistence of Post-traumatic Growth and Post-traumatic Depreciation in the Aftermath of Trauma: Qualitative and Quantitative Narrative Analysis

**DOI:** 10.3389/fpsyg.2019.00687

**Published:** 2019-03-29

**Authors:** Mariusz Zięba, Katarzyna Wiecheć, Joanna Biegańska-Banaś, Wiktoria Mieleszczenko-Kowszewicz

**Affiliations:** ^1^Poznan Faculty of Psychology, SWPS University of Social Sciences and Humanities, Poznań, Poland; ^2^Department of Clinical Psychology, Poznan University of Medical Sciences, Poznań, Poland; ^3^Faculty of Psychology, SWPS University of Social Sciences and Humanities, Warsaw, Poland; ^4^Department of Psychiatry, Jagiellonian University Medical College, Kraków, Poland; ^5^Polish-Japanese Academy of Information Technology, Warsaw, Poland

**Keywords:** post-traumatic growth, post-traumatic depreciation, fundamental assumptions, narrative analysis, LIWC

## Abstract

**Objectives:** Post-traumatic growth (PTG) and post-traumatic depreciation (PTD) can be defined, respectively, as positive and negative changes in the aftermath of trauma. These changes can be assigned to the following domains: personal strength, relating to others, new possibilities, appreciation of life, spiritual and existential change. The aim of this study was to explore the possibility that positive and negative effects of trauma can coexist and explore the categories of effect.

**Methods:** 72 participants were asked to recount their experience of trauma and answer questions about how it had affected their thinking about themselves and the world. Participants’ narratives were analyzed by competent judges and using Linguistic Inquiry and Word Count.

**Results:** The domains in which positive changes were most frequently observed were Personal Strength (26.09%), Relating to Others (24.22%), and Appreciation of Life (21.12%). Negative changes mainly affected Relating to Others (33.33%) and Personal Strength (23.33%). The results were confirmed by quantitative analysis of narratives: participants’ narratives of trauma and its consequences contained more words which expressing positive emotions (1.67%) than negative emotions (0.90%), paired-sample *t*(60) = 9.70, *p* < 0.001. There were correlations between the frequency of words referring to positive emotions and PTG, *r*(62) = 0.39, *p* < 0.01, and between the frequency of words referring to negative emotions and PTG, *r*(62) = 0.23, *p* < 0.05.

**Conclusion:** PTG and PTD can coexist and they can be regarded as outcomes of two separate processes. The study results also suggest that although PTG and PTD can coexist, they may be considered different domains of psychological functioning.

## Introduction

Traumatic events can shatter one’s fundamental assumptions about oneself and the world and have a negative impact on trauma survivors’ sense of self-worth and their beliefs about benevolence and the meaningfulness of the world (Janoff-Bulman, 1992, 2004). Nevertheless, a growing number of studies indicate that having to cope with trauma can result in post-traumatic growth (PTG) (Tedeschi and Calhoun, 2004; Calhoun and Tedeschi, 2006, Joseph and Linley, 2008; Calhoun and Tedeschi, 2013).

In recent years research of the PTG phenomenon typically have used questionnaires that only measure positive changes that may result from trauma, with response options ranging from “no change” to “significant change.” Studies which investigate the negative consequence of trauma have mainly used variables related to post-traumatic disorders, such as post-traumatic stress disorder (PTSD) or affective changes (symptoms of anxiety and depression). There have been few studies measuring negative cognitive changes, especially in fundamental beliefs. Baker et al. (2008) proposed a “post-traumatic depreciation” (PTD) construct, an inverse of PTG referring to negative changes in the same domains: changes to sense self, changes to one’s perception of one’s relationships with others and changes to philosophy of life.

Research show that PTG and PTD should be considered as independent constructs. They are not or slightly correlated, and they have different predictors and outcomes (Baker et al., 2008; Cann et al., 2010; Barrington and Shakespeare-Finch, 2013; Kroemeke et al., 2017). For example, both deliberative and intrusive ruminations predict PTD, but only deliberative ruminations predict PTG (Allbaugh et al., 2016); PTD, but not PTG, relates to distress, depression, anxiety and satisfaction with life (Barrington and Shakespeare-Finch, 2013); problem-focused and positive emotion-focused coping predict PTG, but negative emotion and avoidance-focused coping predict PTD (Kroemeke et al., 2017).

Most of the research on positive or both positive and negative changes after trauma use quantitative measures (Tedeschi et al., 2018). It is worth noting that original items of first and mostly used questionnaire to measure PTG (PTGI, Tedeschi and Calhoun, 1996) are quotes and adapted quotes from interviewees who had been trauma survivors. Likewise the Change in Outlook Questionnaire (Joseph et al., 1993), which measure both positive and negative post-trauma changes, was designed based on the interviews with survivors of disasters. Qualitative methods are more difficult to use than questionnaires, but they can help to provide more complex explanations of different individual and situational factors affecting the experience of PTG and PTD (Tedeschi et al., 2018).

The coexistence of PTD and PTG found in previous quantitative studies can be explained by the fact that positive and negative changes may occur at the same time in different domains, for example growth of personal strength and concurrently weakening trust in other people. It is more difficult to explain simultaneous changes in the same domain. The qualitative methods seem to be useful for this purpose. For example, the results of the analysis of interviews with the athlete who recovery from traumatic and disabling injury (Day and Wadey, 2016) could explain coexistent of synchronous growth and depreciation in the same domain: developing new relationships with other athletes negatively affected existing relationships.

The aim of this study was to verify with the use of qualitative analysis previous findings about coexistence of PTG and PTD in the same and different domains, and associations between PTG, PTD, depression, anxiety and ruminations. An additional purpose was to examine whether PTG and PTD relate to negative and positive affect in the narratives of people who survived the trauma.

## Methods

Students of master in psychology graduate program were invited to participate in narrative interview about important life events. Participants had received information about procedure and the Informed Consent Form before the interview and they could withdraw from the study at any time. The study was approved by the university ethical commission. The procedure included demographic questions, questions about health status and traumatic experiences in recent years. The participants were asked the following question:

A trauma can be defined as an event that a person witnessed, or was confronted with that involved actual or threatened death or serious injury, or a threat to the physical integrity of the self or others and responded to with intense fear, helplessness, or horror. Have you experienced this type of event in the last 12 months?

Seventy-eight out of 300 participants reported that they had experienced trauma in the last 12 months: serious personal illness (18.5%), illness of a significant other (15.4%), death of a significant other (12.8%), being a victim of a crime such as robbery or mugging (8.7%) or accident that led to personal injury (8.2%) or injury of a significant other (7.1%). Seventy-two people consented to participate in the procedure presented below. After excluding incomplete data the sample comprised 61 individuals (53 women) aged from 21 to 56 years (*M* = 32.92, *SD* = 8.38).

### Questionnaires

#### Symptoms of Anxiety and Depression

We measured the presence and severity of anxiety and depression symptoms in the past week using the Hospital Anxiety Depression Scale (HADS; Zigmond and Snaith, 1983), a self-rating scale consisting of 2 subscales: HADS-A, comprising 7 anxiety-related items (e.g.: “I get sudden feelings of panic”) and HADS-D, comprising 7 depression-related items (e.g., “I feel as if I am slowed down”). Responses were given using a 0 to 3 scale.

#### Intrusive and Deliberate Rumination

The extent of intrusive and deliberate rumination in the aftermath of the traumatic event was assessed using the Event-Related Rumination Inventory (ERRI; Cann et al., 2011), which comprises 10 items measuring intrusive rumination (e.g., “Thoughts about the event came to mind and I could not stop thinking about them”) and 10 items measuring deliberate rumination (e.g., “I thought about whether I could find meaning from my experience”) to which responses were given using a 4-point scale (0 = not at all to 3 = often).

### Interview

Next a structured interview was conducted by a qualified psychologist. The participants were asked to recount the traumatic event they had experienced and answer questions about the impact of the described event on different areas of life, like: changes in the perception of oneself, others, the world and the future, as well as the impact of own behavior, emotions, physical sensations and relationships with others.

Interviews typically lasted about 35 to 45 minutes. They were audio-recorded and transcribed. The length of transcriptions ranged from 298 to 11972 words (*M* = 3335, *SD* = 2212).

### Narrative Analysis

This analysis was conducted by the second author and the results were discussed with the research team. MAXQDA 11 software was used to process the data.

The analysis of participants’ narratives was divided into two phases, descriptive and interpretative (Murray, 2008). The first, descriptive stage involved immersion in the data - reading and re-reading the transcripts - to identify structure and content of the narratives. At this stage all fragments of the interviews were coded by themes related to negative and positive post-traumatic changes in beliefs about oneself or the world-view. The second, interpretative stage was required to connect the themes of the narratives with the relevant theoretical literature (Murray, 2008).

We also used computerized text analysis to identify the negative and positive features of the participants’ narratives. Transcripts of interview responses (i.e., not including the interviewer’s words) were analyzed using Linguistic Inquiry Word Count (LIWC; Tausczik and Pennebaker, 2010) supported by a Polish dictionary (Szymczyk et al., 2012).

## Results

Negative and positive categories of posttraumatic changes identified in the first stage of the study were found to be substantially related to PTG domains described by Calhoun and Tedeschi (2006). The categories are as follows: Personal Strength, Relating to Others, New Possibilities, Appreciation of Life, Spiritual and Existential Change.

**FIGURE 1:**
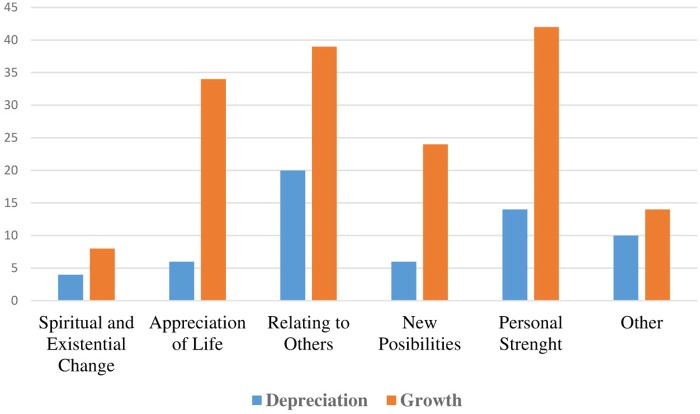
Number of study participants indicating that they had experienced specific categories of post-traumatic depreciation and post-traumatic growth.

The frequency of changes in specific PTG and PTD area are shown on Figure 1.

Most frequently positive changes were observed in Personal Strength (increased sense of self-reliance, strength and confidence), Relating to Others (experience of positive changes in relationships), and Appreciation of Life (greater appreciation for all the things that life has to offer), and negative changes in Relating to Others. Analysis at the level of the individual showed that negative and positive changes often co-occur: 28 people reported that they had experienced both PTG and PTD and 17 indicated similar, fairly high levels of both PTG and PTD. Seven persons mentioned only or predominantly negative changes and 29 persons only or predominantly positive changes. Six respondents did not report any changes indicative of PTG or PTD.

Some people who reported both PTG and PTD had experienced positive and negative changes in different categories. Positive changes in one of the domains of PTG, e.g., personal strength, were accompanied by negative changes in other domains, e.g., relations with other people.

This is illustrated by a quotation from OB125:

I mean, I certainly felt much stronger, right? Much stronger (...). However, here, kind of from a higher level, from a higher perspective, I learned a bit about how to sort of manage people. And I learnt to be a bit better at setting boundaries and how not to be a welcome-mat (...) I think I feel stronger, right? I know I can. {Mhm.} That I can do much more than I thought I could, right?

[...] I will certainly be more careful. When it comes to my contact with people. And trust in people. It taught me to be really careful. So in general when I talk to someone, the me from a year ago and the me of today are two different people.

Fourteen participants (19.4%) reported experiencing both positive and negative changes in the same domain. For example, as a result of trauma individuals experienced increased sense of agency in the professional area and decreased sense of agency in other life areas, or improved relations with some close relatives and worsened relations with others, as in the case of OB28: “Certainly, relations with my partner’s family have been negatively affected. However, it seems to me that it has had a positive impact on the relationship between us, yes, on our relationship. And it seems to me that it has strengthened our relationship, right?”

The results of the computerized text analysis, presented in Table 1, indicate participants’ narratives of trauma and its consequences contained more words which expressing positive emotions (1.67%) than negative emotions (0.90%), paired-sample *t*(60) = 9.70, *p* < 0.001. Correlation analysis showed that the frequency of words referring to positive emotions was positively related to PTG; there was also a weaker relationship between the PTG and the frequency of references to negative emotions. The correlation coefficients were presented in the Table 1.

**Table 1:** Means, standard deviations and Spearman’s correlation coefficients for the study variables.

	*M*	*SD*	*1*	*2*	*3*	*4*	*5*	*6*	*7*
(1) Depression (HADS)	3.44	4.56							
(2) Anxiety (HADS)	6.19	4.25	0.60***						
(3) Intrusive ruminations (ERRI)	26.30	7.94	0.32**	0.55***					
(4) Deliberative ruminations (ERRI)	27.83	6.27	0.01	0.36**	0.47***				
(5) Positive emotions (LIWC)	1.67	0.58	-0.14	-0.07	0.09	0.09			
(6) Negative emotions (LIWC)	0.90	0.37	-0.11	0.13	0.15	0.22	0.13		
(7) Post-traumatic growth (interview)	5.78	4.90	-0.26*	-0.18	0.04	0.21	0.39**	0.23*	
(8) Post-traumatic depreciation (interview)	1.58	2.42	0.48***	0.34**	0.33*	-0.04	-0.16	-0.01	-0.28*


*HADS, Hospital Anxiety Depression Scale; ERRI, Event-Related Rumination Inventory; LIWC, Linguistic Inquiry Word Count. ^∗^*p* < 0.05, ^∗∗^*p* < 0.01, and ^∗∗∗^*p* < 0.001.*

There was a negative relationship between PTG and PTD, as well as negative relationship between PTG and symptoms of anxiety and depression and a positive relationship between PTD and depression. There were no age effects.

## Discussion

Our analysis of the narratives of trauma survivors has added to knowledge about the coexistence of PTG and PTD. The results indicate that both constructs can co-exist, which corroborates research by Purc-Stephenson et al. (2015) and Kroemeke et al. (2017). The negative correlation between the numbers of positive and negative changes reported by participants also seems partially to confirm the view that PTG and PTD are outcomes of separate processes (Cann et al., 2010; Forgeard, 2013).

Thirty eight (53%) participants were in the high PTG-low PTD group in the study. Their narratives contained more references to positive emotions than to negative emotions. This result supports previous studies showing that there is a greater number of people who report experiencing more PTG than PTD than those who report experiencing mostly negative changes in the aftermath of trauma (e.g., Chen and Wu, 2017; Kroemeke et al., 2017; Cao et al., 2018).

It is worth noting that the narrative analysis highlighted that participants fairly frequently report the co-occurrence of PTG and PTD, thus providing further evidence that positive and negative consequences of trauma can co-occur. The results also suggest that although PTG and PTD can co-occur, they represent different domains of psychological functioning.

The use of narrative methodology in PTG and PTD research makes it possible to construct a more complex picture than is provided by questionnaire-based studies, which are much more frequent. It would be worth carrying out further research with larger and more diverse groups (including the type and severity of trauma) in order to investigate situational and individual factors that influence whether positive changes in the aftermath of trauma are accompanied by negative changes or not, and to explore the relationships between particular domains of PTG and PTD.

Limitations of the study is the research group based on psychology students as they are very feminized group and, due to the specificity of their studies and knowledge of various psychological mechanisms, may differ in building the narrative and drawing conclusions of the experiences from people in the whole population. The next step should be to expand the research group to a more diverse group of adults. Going beyond cross sectional studies in order to elucidate the longitudinal associations between PTG and PTD should be also considered.

## Ethics Statement

This study was carried out in accordance with the recommendations of SWPS University of Social Sciences and Humanities Ethics Committee. All participants gave written informed consent in accordance with the Declaration of Helsinki. The protocol was approved by the University Ethics Committee.

## Author Contributions

MZ lead the project. KW gathered the data and was instrumental in developing the article, the topic area and interpreting the data. JB-B was instrumental in developing the article, the topic area and interpreting the data. WM-K was instrumental in interpreting the data using computerized text analysis.

## Conflict of Interest Statement

The authors declare that the research was conducted in the absence of any commercial or financial relationships that could be construed as a potential conflict of interest.
